# An investigation of the effects of different text formats on middle school students’ reading comprehension performance

**DOI:** 10.1371/journal.pone.0331786

**Published:** 2025-10-01

**Authors:** Betül Koparan

**Affiliations:** Department of Turkish Language Education, Faculty of Education, Akdeniz University, Antalya, Türkiye; Zhejiang Normal University, CHINA

## Abstract

This study is a quasi-experimental research that aims to compare the reading comprehension levels of students who read texts in textbooks through printed, digital, and augmented reality (AR)-supported formats. The sample of the study consisted of 150 students aged 11–12 who were enrolled in schools in Turkey. The participating students were randomly assigned to three equal groups (print, digital, augmented reality). During the data collection process, reading comprehension was assessed in five dimensions (literal comprehension, reorganization, inferential comprehension, evaluation, and appreciation) based on Barrett’s Taxonomy. In order to determine the students’ reading comprehension achievement levels, a “reading comprehension achievement test” was used. Initially, reading activities using the same printed texts were conducted with all three groups. Afterwards, a pre-test was administered. Following a five-week break, different reading activities were conducted with the three groups during the post-test phase. Intra-group comparisons of pre-test and post-test data were analyzed using the paired-samples t-test. ANCOVA analysis was used to test whether the differences in post-test reading comprehension scores between the groups were statistically significant. The results showed that the AR-supported reading activity improved students’ reading comprehension performance (p < .05, η^2^ = .388). The same trend was observed in all sub-dimensions of reading comprehension (literal comprehension p < .05, η^2^ = .140; reorganization p < .05, η^2^ = .217; inferential comprehension p < .05, η^2^ = .322; evaluation p < .05, η^2^ = .225; appreciation p < .05, η^2^ = .327). Therefore, it can be concluded that augmented reality books enhance the reading comprehension performance of children aged 11–12. On the other hand, there was no significant difference in reading comprehension performance between traditional reading and screen-based reading (p = .542). These findings indicate that AR content is more effective in improving students’ reading comprehension compared to printed and digital texts when reading storybooks. Consequently, the present research provides insights into the effects of different text formats on middle school students’ reading comprehension performance.

## 1. Introduction

Reading is an active comprehension process that aims for students to go beyond sentences and grasp the intended meanings of a given text [1]. In this context, the primary goal of reading is understanding, that is, constructing meaning [2]. Additionally, reading is defined as a fundamental tool for communication and learning processes, as well as an indispensable skill for individuals to achieve personal and social success [3].

It can be stated that students’ reading comprehension skills have a significant impact on various aspects, such as learning, problem-solving, cognitive development, and academic achievement [4]. An effective reading process and interactive reading books play a critical role in motivating children to read and supporting cognitive components such as thinking, explaining, understanding, and remembering [5]. In this context, the variety of reading texts has increased in recent years with the rise of digital tools that can assist students in improving their reading comprehension. In addition to printed books, books available on digital platforms offer readers a wide range of content in various formats. This variety not only makes reading more accessible but also allows readers to access customized content tailored to their interests and needs [6]. Furthermore, texts that include visual and auditory elements enrich learning processes and make the reading experience more interactive and effective [7,8]. It has also been found that different types of reading texts contribute to developing readers’ thinking and comprehension skills [9]. In this framework, studies on reading comprehension skills indicate that the technologies used can positively affect reading comprehension [10,11].

When the relevant literature is examined, it is evident that many studies have investigated the impact of traditional reading on reading comprehension [12–14]. However, with the emergence of digital reading, new research in this area has become necessary. Existing studies have focused on topics such as readers’ digital reading habits and the effects of digital environments on comprehension processes [15–19].

Subsequently, studies comparing traditional and screen reading have been conducted to evaluate their respective impacts on individuals’ reading comprehension skills [20–24]. The benefits of digital books identified in these studies include fostering habits such as skimming and scanning digital content [25,26], enhancing reading comprehension skills [4,27,28], and improving phonological awareness [29]. Furthermore, a study by Siegenthaler et al. [30] found that participants exhibited more extended periods of focus when reading on digital devices than printed books.

However, some studies have indicated that students reading digital texts tend to skim and scan rather than read thoroughly, negatively affecting comprehension and attention to detail [25,26]. Additionally, when asked to answer more challenging questions requiring attention to specific details, students who read digital texts performed worse than those who read printed texts [31]. Other findings suggest that screen reading may cause eye strain [32], and digital environments may disrupt concentration, leading to more superficial information processing [33]. However, whether screen reading is better or worse than traditional reading remains unclear [32,34]. In this context, it can be argued that more detailed studies are needed to explore the effects of various reading environments, diversified by technological advancements, on students’ reading comprehension.

In today’s world, digitalization and technological innovations have significantly transformed the types of reading materials and reading processes. This transformation not only provides students with diverse and innovative tools to develop their reading skills but also creates important opportunities in the field of education. In particular, augmented reality (AR) technology adds a more interactive dimension to students’ reading experiences by integrating traditional reading with digital elements. In this context, AR storybooks offer an innovative reading experience that combines the advantages of both printed books and digital formats, thereby contributing to students’ reading comprehension skills [35,36]. AR is a novel technology that allows users to perceive virtual objects integrated with or superimposed on real-world objects [37–40]. Given its features, the use of AR in education is anticipated to bring numerous benefits [41–46]. Studies in this context have shown that AR applications make the learning process more engaging [47–49], increase students’ motivation [50–54], and are effective in teaching abstract and complex topics [51], ultimately positively influencing students’ academic performance [51,55–59].

The potential of AR in educational settings has been explored under various topics [60–62]. One of these topics is AR storybooks [63], which stand out as a new technology offering a combined experience of printed and digital reading [64,65]. Various studies have been conducted in this context, addressing different study groups and contexts [35,66–73]. However, the limited number of studies on how AR books affect students’ reading comprehension skills highlights the need to examine the effectiveness of this technology in education in more detail [61,65,66]. Furthermore, many studies have only compared AR and traditional reading [74], highlighting the need for research comparing different reading tools’ effects on reading comprehension. At this point, the study aims to make a significant contribution to the existing literature by examining the effects of AR storybooks on students’ reading comprehension skills through a comparative analysis of different reading materials. Therefore, there is a need for a more detailed understanding of how these reading materials affect participants’ reading comprehension performance.

Although there has been an increase in the number of studies in the literature examining the effects of AR-supported books on students’ reading comprehension, it is observed that these effects are mostly evaluated based on overall achievement scores, while more detailed analyses related to the reading process remain limited [66,75]. Furthermore, most research on how the digitalization process transforms reading habits tends to focus solely on the differences between traditional and digital reading types, whereas studies that comparatively examine the pedagogical impacts of hybrid technologies like AR remain scarce [62,65].

In this context, the present study comparatively investigates the impact of three different reading environments—traditional, digital, and AR-supported books—on students’ reading comprehension performance, and explores in depth how each type of reading differs at the cognitive level. Conducted with middle school students in the context of Turkey, the research holds the potential to offer generalizable insights both specific to national educational settings and to globally digitalized learning environments.

In this respect, the study aims to make meaningful contributions to the literature not only in terms of pedagogy but also in relation to the implementation of educational technologies. Moreover, the findings obtained from this study are of a nature that can guide teachers and educational material developers in selecting effective reading materials for students.

## 2. Review of the literature

### 2.1. Reading comprehension

Reading comprehension is defined as an interactive and critical process that requires understanding and accurately interpreting the information presented in texts [76,77]. Once learned, it is a skill that tends to remain relatively stable in individuals [78]. However, it has been found that children who experience difficulties in reading comprehension often struggle throughout their school years and exhibit low levels of engagement in educational settings [79]. In this context, acquiring and developing reading comprehension skills is crucial for students’ success in both personal and academic life [80].

Books are one of the most fundamental tools that can be used to develop reading comprehension skills. Storybooks and picture books motivate children to read and support cognitive components such as thinking, explaining, understanding, and remembering [5]. Similarly, the National Reading Panel [81], which evaluated the findings of studies on the reading comprehension process, revealed that enjoyable early reading experiences enhance children’s attitudes toward reading [82,83] and that children generally prefer engaging reading-related activities [84,85]. Therefore, preparing reading materials that can capture children’s interest is very important.

With the rapid advancements in digital technology, various reading materials have emerged. Studies in the literature have primarily focused on printed texts and digital texts. When examining research on the effectiveness of digital or printed formats in the context of reading comprehension skills, some studies have found results in favour of the printed format [86–89]. On the other hand, some studies have shown that participants reading digital texts performed better [90–92]. Meanwhile, other studies have revealed no significant differences between groups, regardless of whether the reading material was in a printed or digital format, indicating similar performance in reading comprehension tasks [93–96]. However, a significant portion of the research [97–101], along with meta-analytical studies [102–104], has demonstrated that individuals’ reading comprehension performance is higher with printed texts compared to digital texts. Despite these findings, it can be said that the results of studies in the relevant literature are inconsistent.

With advancements in digital technologies, digital texts have also diversified [105–107]. Different formats, such as electronic storybooks and interactive texts, are now widely used. In recent years, AR storybooks have emerged as an emerging format that combines printed and digital books [108,109]. Studies have shown that AR storybooks act as a bridge between real and virtual worlds by enabling readers to dynamically interact with virtual content in a real environment [65,69,110,111]. They have been found to enhance students’ academic performance [112], provide engaging and entertaining content, and promote active reading, thereby improving reading performance [6,113–115]. Additionally, AR storybooks encourage touch interaction during content exploration, providing a more interactive space for storytelling [116]. These features have led to participants exhibiting positive attitudes toward using AR books [117,118]. On the other hand, some studies highlight drawbacks, such as the potential for AR reading to be cognitively demanding and distracting if not used properly [119,120], as well as concerns related to excessive screen use [97,121]. Furthermore, there is no consensus on whether AR is more effective than printed books in improving reading comprehension [122], and there is insufficient discussion on how AR books should be designed and implemented [123]. Indeed, the literature reveals that research on the impact of AR on students’ reading comprehension is quite limited [54,61,65,66,97,121,124]. Therefore, it can be stated that there is a need for in-depth studies on the effects of AR compared to printed and digital books on reading comprehension. Notably, no study has been found in the literature evaluating these three formats. In the present study, Barrett’s taxonomy was used to obtain more detailed results regarding reading comprehension.

### 2.2. Barrett taxonomy

Barrett’s taxonomy, developed to measure reading skills, was first introduced in the “Innovation and Change in Reading Instruction” published by the American National Educational Research Association [125]. The individual who elaborated on Barrett’s taxonomy was Theodore Clymer [126]. Clymer discussed Thomas C. Barrett’s unpublished paper titled “Taxonomy of Cognitive and Affective Dimensions of Reading Comprehension” with his permission and introduced this taxonomy in his article “What is ‘Reading’: Some Current Concepts” [126].

Barrett’s taxonomy [127] was specifically designed to assess students’ comprehension skills in the field of reading. It was initially used by teachers in classrooms to classify and prepare comprehension questions [126]. In Barrett’s taxonomy, reading comprehension is divided into five categories: (1) literal comprehension, (2) reorganization, (3) inferential comprehension, (4) evaluation, and (5) appreciation. For each category, Barrett provided examples of specific tasks and listed objectives (competency areas) that teachers could use [126,128]. The literal comprehension level focuses on understanding explicitly stated information and ideas in the text. The reorganization level involves accurately understanding the text by using information from various text sections and establishing connections between them [129]. Inferential comprehension, another type of understanding, requires readers to make inferences by relying on their background knowledge and attempting to interpret the implicit information in the text [127]. The evaluation level involves questioning a reading text using predetermined criteria and reaching a judgment based on the findings [130]. Finally, the appreciation level encompasses all cognitive dimensions of comprehension and concerns texts’ psychological and aesthetic effects on readers [129].

### 2.3. *The current study*

This study examined the effect of printed, digital, and augmented reality-supported texts on students’ reading comprehension performance. In this context, the study aimed to answer the following research questions:

1. What is the difference in reading comprehension scores among groups reading printed, digital, and augmented reality-supported texts?
2. What is the difference in scores for literal comprehension, reorganization, inferential comprehension, evaluation, and appreciation among groups reading printed, digital, and augmented reality-supported texts?

## 3. Methodology

### 3.1. Participants

The participants consisted of 150 students, aged between 11 and 12, who were studying at a middle school in Antalya, Turkey. The students were randomly divided into three groups of 50 each: Group 1 (23 females and 27 males), Group 2 (26 females and 24 males), and Group 3 (27 females and 23 males). The sample size was determined using the G*Power software, and a power analysis was conducted. Power analysis was conducted based on a specified effect size (d = 0.8), alpha level (α = 0.05), and power (1 – β = .95) parameters. This analysis aimed to assess the likelihood of obtaining statistically significant results from the tests conducted on the current sample. Participants were selected only from among students who voluntarily agreed to participate in the reading activities. Data collection began after obtaining ethical approvals and informed consent forms from the students’ families. Information about the participants is presented in Table 1 below.

**Table 1:** Information about the participants.

	Group 1	Group 2	Group 3
Male	27	24	23
Female	23	26	27
**Have a technological device**			
*Have a smartphone*	36	35	37
*Have a computer*	46	48	45
*Have a tablet*	35	31	34
**Purpose of use of digital equipment**			
*Game playing*	45	43	47
*Communication*	27	31	29
*Watching videos/movies*	44	43	46
*Studying*	36	41	39
*Taking photos*	10	8	5

### 3.1. Printed and digital book

The texts used in the present study were taken from the 6th-grade native language textbook used nationwide. A total of three texts were utilized: “Aziz Sancar, Mustafa Kemal Zafer Yolunda (Mustafa Kemal on the Road to Victory), and İklim Değişikliği ve Toplum (Climate Change and Society)”. These texts were selected due to their appropriateness for the age level and their potential to provide insights into the usability of different technologies in school settings and textbooks. Therefore, these texts were chosen from the native language textbooks, whose printed versions are distributed to all students nationwide.

For the printed text reading sessions, the texts were read directly from the textbooks. On the other hand, the digital book version was identical to the printed book and was the digital textbook published online by the Ministry of National Education. This digital book was accessed on a computer for the digital reading activities. Thus, there was no difference in content between the printed and digital books.

### 3.2. The augmented reality storybook

**Fig 1:**
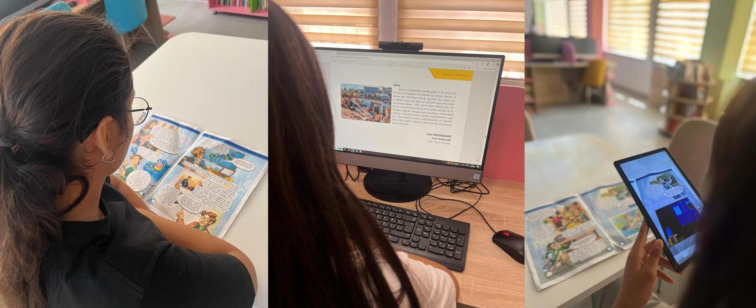
Reading Activities.

In the present study, three texts (Aziz Sancar, Mustafa Kemal Zafer Yolunda, and İklim Değişikliği ve Toplum) from the native language textbooks used nationwide were utilized. The Ministry of National Education published these texts in printed and digital formats, which are already used in schools. To create the augmented reality (AR)-supported texts for the study, the Roar AR application was employed. This application allows the AR content embedded in the images within the text to be triggered without using QR codes or markers. First, AR content was developed, and the AR content-image matching was completed via the application’s web interface. To develop the AR content, the design principles established in the study by Şimşek et al. [131] were followed. Their study emphasized presenting multimedia content in AR-supported texts in harmony and balance with the text to support students’ cognitive load and reading comprehension levels. Based on these principles, AR content was prepared to enhance understanding while maintaining alignment with the text. The duration of the AR experiences ranged between 24–27 seconds. Additionally, the videos prepared as AR content included audio narrations of the relevant text sections, ensuring that the content aligned with the corresponding portions of the text. Care was taken to ensure that the AR content was seamlessly integrated into the flow of the printed text, maintaining its coherence and contributing to students’ comprehension. The prepared videos were matched with the images in the texts using the Roar AR application’s online platform. As a result, when the application was opened on a mobile device, the camera was activated, and the AR content was triggered upon detecting the corresponding image. Notably, the application displayed the video and the other sections of the text visible within the camera’s frame. This feature was essential to ensure students remained engaged with the text and did not lose focus. Ultimately, during the reading activities, students used printed texts and the Roar AR application through tablets without requiring computer or coding knowledge. The visuals related to the reading activities conducted in the study are presented in Fig 1.

### 3.3. Measures

#### 3.3.1. Reading comprehension achievement test.

To determine students’ reading comprehension achievement levels, the “Reading Comprehension Achievement Test” developed by Şimşek and Direkci [74] based on the texts used in the present study was utilized. The test consists of 23 items, 11 of which are multiple-choice and 12 open-ended. The difficulty levels of the items range from 0.48 to 0.73, while their discrimination indices range from 0.46 to 0.81. The achievement test was administered to 150 participants as part of the study. According to the test results, the KR-20 reliability coefficient was calculated as 0.764. This coefficient indicates that the results obtained from the achievement test are reliable [132]. In the multiple-choice questions of the achievement test, incorrect answers were scored as 0, and correct answers were scored as 1. For the open-ended questions, incorrect answers were scored as 0, partially correct answers as 1, and entirely correct answers as 2. Based on the challenges of evaluating open-ended questions related to readers’ creative and critical thinking skills, students were informed that their answers would only be correct if they were relevant to the text. Three experts conducted the evaluation, and Kendall’s W coefficient of concordance was calculated. This coefficient was found to be 0.937 for the pre-test and 0.959 for the post-test. Examples of questions from the reading comprehension test are as follows:

1. *Which of the following is not one of the topics Aziz Sancar worked on? (Literal Comprehension)*
2. *What do you think about the reality of the climate change events described in the text you read? Explain your answer with justification. (Evaluation)*
3. *If a group of migrants forced to relocate due to climate change wanted to come to your country, how would you respond? Explain your reasoning. (Response)*

### 3.4. Procedures

This study was conducted in accordance with ethical principles and received approval from the Ethics Committee of Akdeniz University (Approval No: 1007503, dated 10.09.2024). Additionally, permission was obtained from the Provincial Directorate of National Education to carry out the research process in schools. Written consent forms were also collected from both students and their parents to include the participants in the study. Following the completion of all necessary permissions, meetings were held with the administrators of the school where the application would be conducted, and the field implementation process was initiated. Subsequently, meetings were arranged with the teachers responsible for the implementation classes, and they were informed about the study. Additionally, the augmented reality (AR) application was introduced to the teachers, and its use in reading activities was demonstrated through hands-on sessions. This was because teachers in the classroom, alongside the researcher, were requested during the implementation process to ensure that students felt more comfortable. The researcher carried out the implementation process. Teachers remained in the classroom only to assist with tablet usage and to communicate with students if necessary. The data collection process in schools started on 1.10.2024 and ended on 18.11.2024. This study is a quasi-experimental design with a pretest-posttest control group, aiming to examine the changes in participants before and after the intervention. During the pre-test phase of the study, all groups participated in reading activities using only printed texts. Afterwards, the achievement test was administered, and pre-test data were collected. A five-week interval was given between the pre-test and post-test phases, during which students followed their regular lesson schedules. Following this period, the three groups participated in different reading activities to collect post-test data. The first group read printed texts, while the second group read digital texts via computers. The third group read printed texts and experienced AR content using tablets at relevant text points. Subsequently, the achievement test was administered to all three groups, and post-test data were collected. During the test sessions, students were informed that they should feel at ease while answering and that the test results would not be used for evaluative purposes. After completing the pre-test and post-test phases, the analysis process began.

## 4. Results

### Research Question 1: The effect of reading printed, digital, and augmented reality-supported texts on the reading comprehension scores of different groups

The study initially conducted reading activities using the same printed texts with all three groups. Following this, a pre-test was administered. In the post-test phase, the three groups engaged in reading activities in different formats. The results indicated that augmented reality (AR)-supported reading activities improved students’ reading comprehension performance. The same results were observed across the sub-dimensions of reading comprehension (literal comprehension, reorganization, inferential comprehension, evaluation, appreciation). There was no statistically significant difference in comprehension performance between students who participated in printed text reading activities and those engaged in screen-based reading activities. Table 2 presents each group’s pre-test and post-test means, standard deviations, and analysis results.

**Table 2:** Mean and standard deviation for reading comprehension.

			Pre-test		Post-test			
	Group	N	Mean	Sd	Mean	Sd	*t*	*p*
Total score	Group 1	50	17.200	5.387	18.600	4.440	−1.290	.203
	Group 2	50	16.640	4.471	17.340	4.264	−.777	.441
	Group 3	50	18.180	4.359	25.380	4.453	−27.017	.000
Literal comprehension	Group 1	50	3.740	1.601	3.840	1.390	−.338	.737
	Group 2	50	3.520	1.568	3.920	1.275	−1.353	.182
	Group 3	50	3.920	1.482	5.020	1.285	−5.723	.000
Reorganization	Group 1	50	2.820	1.189	3.160	1.201	−1.426	.160
	Group 2	50	2.680	1.202	2.820	1.003	−.590	.558
	Group 3	50	3.020	1.133	4.040	.668	−5.993	.000
Inferential comprehension	Group 1	50	4.600	1.456	4.760	1.450	−.531	.598
	Group 2	50	4.540	1.417	4.500	1.460	.158	.875
	Group 3	50	4.960	1.428	6.640	1.156	−10.487	.000
Evaluation	Group 1	50	2.940	1.268	3.420	1.213	−1.841	.072
	Group 2	50	3.020	1.377	3.180	1.206	−.599	.552
	Group 3	50	3.140	1.324	4.620	1.104	−7.308	.000
Appreciation	Group 1	50	3.100	1.717	3.420	1.196	−1.008	.318
	Group 2	50	2.880	1.319	2.920	1.291	−.158	.875
	Group 3	50	3.140	1.030	5.060	1.448	−9.716	.000

Since the pre-test and post-test data obtained in the study exhibited a normal distribution, a paired-sample t-test was conducted. Following the intervention, Group-1’s total test score increased from 17.2 to 18.6 (t = −1.290, p > .05), literal comprehension score increased from 3.74 to 3.84 (t = −.338, p > .05), reorganization score increased from 2.82 to 3.16 (t = −1.426, p > .05), inferential comprehension score increased from 4.6 to 4.76 (t = −.531, p > .05), evaluation score increased from 2.94 to 3.42 (t = −1.841, p > .05), and appreciation score increased from 3.1 to 3.42 (t = −1.008, p > .05). These results indicate that there was no significant difference in the reading comprehension performance of students who participated in printed text reading activities before the pre-test and post-test phases. According to the analysis results, Group-2’s total test score increased from 16.64 to 17.34 (t = −.777, p > .05), literal comprehension score increased from 3.52 to 3.92 (t = −1.353, p > .05), reorganization score increased from 2.68 to 2.82 (t = −.590, p > .05), evaluation score increased from 3.02 to 3.18 (t = −.599, p > .05), and appreciation score increased from 2.88 to 2.92 (t = −.158, p > .05). However, inferential comprehension score decreased slightly from 4.54 to 4.5 (t = .158, p > .05). These results suggest that screen-based reading activities did not create a significant difference in students’ reading comprehension performance. The results were consistent across other sub-dimensions.

### Research question 2: The effect of reading printed, digital, and augmented reality-supported texts on students’ scores in literal comprehension, reorganization, inferential comprehension, evaluation, and response

The pre-test and post-test results for Group 3 differed significantly from those of the other groups. According to the analysis results, Group 3’s total test score increased from 18.18 to 25.38 (t = −27.017, p < .05), literal comprehension score increased from 3.92 to 5.02 (t = −5.723, p < .05), reorganization score increased from 3.02 to 4.04 (t = −5.993, p < .05), inferential comprehension score increased from 4.96 to 6.64 (t = −10.487, p < .05), evaluation score increased from 3.14 to 4.62 (t = −7.308, p < .05), and appreciation score increased from 3.14 to 5.06 (t = −9.716, p < .05). These results indicate that the augmented reality (AR) intervention significantly enhanced students’ reading comprehension performance. This finding was consistent across all sub-dimensions of reading comprehension. When the effect size was examined, a large effect size was identified for total score (d = 1.63), reorganization (d = 1.08), inferential comprehension (d = 1.26), evaluation (d = 1.20), and appreciation (d = 1.5). In contrast, a medium effect size was found for literal comprehension (d = .78) [133].

## 3. Evaluation of post-test differences between the three groups

ANCOVA analysis was conducted to test whether the differences in post-test reading comprehension scores among the groups were statistically significant. The adjusted post-test mean scores based on pre-test scores and the analysis results are presented in Table 3.

**Table 3:** ANCOVA result of the post scores on students’ reading comprehension performance.

	Group	N	Mean	Sd	Adjusted mean	Std. error	*F*	*η2*
Total score	Group 1	50	18.600	4.440	18.623	.612	46.209*	.388
	Group 2	50	17.340	4.264	17.457	.614		
	Group 3	50	25.380	4.453	25.239	.615		
Literal comprehension	Group 1	50	3.840	1.390	3.838	.185	11.862*	.140
	Group 2	50	3.920	1.275	3.946	.185		
	Group 3	50	5.020	1.285	4.995	.185		
Reorganization	Group 1	50	3.160	1.201	3.160	.139	20.173*	.217
	Group 2	50	2.820	1.003	2.819	.140		
	Group 3	50	4.040	.668	4.041	.140		
Inferential comprehension	Group 1	50	4.760	1.450	4.782	.188	34.684*	.322
	Group 2	50	4.500	1.460	4.535	.189		
	Group 3	50	6.640	1.156	6.583	.189		
Evaluation	Group 1	50	3.420	1.213	3.424	.167	21.170*	.225
	Group 2	50	3.180	1.206	3.181	.167		
	Group 3	50	4.620	1.104	4.616	.167		
Appreciation	Group 1	50	3.420	1.196	3.416	.186	35.489*	.327
	Group 2	50	2.920	1.291	2.930	.187		
	Group 3	50	5.060	1.448	5.054	.187		

*p < .05

The results demonstrated that the intervention had a significant overall effect on reading comprehension [F(2, 146) = 46.209, p < .05, η^2^ = .388]. When comparing the groups individually, a significant difference in reading comprehension performance was observed between Group-3 and Group-1 (p = .000) and between Group-3 and Group-2 (p = .000). However, no significant difference was found between Group-1 and Group-2 (p = .542). Therefore, it can be concluded that the augmented reality (AR) intervention provided more significant support for children’s reading comprehension performance compared to reading printed texts or digital texts on a screen. Additionally, although there was no statistically significant difference, children who read printed texts scored higher than those who read from a screen.

## 4. Intergroup comparisons based on sub-dimensions

Although no statistically significant difference was found between the two groups, it was observed that children who read printed texts scored higher than those who read on digital screens. This trend was consistent across other dimensions of reading comprehension. Significant differences in favour of the AR intervention were also found in the sub-dimensions of literal comprehension (Group-1 and Group-2: p = .069; Group-1 and Group-3: p = .000; Group-2 and Group-3: p = .000), reorganization (Group-1 and Group-2: p = .259; Group-1 and Group-3: p = .000; Group-2 and Group-3: p = .000), inferential comprehension (Group-1 and Group-2: p = 1.000; Group-1 and Group-3: p = .000; Group-2 and Group-3: p = .000), evaluation (Group-1 and Group-2: p = .914; Group-1 and Group-3: p = .000; Group-2 and Group-3: p = .000), and appreciation (Group-1 and Group-2: p = .203; Group-1 and Group-3: p = .000; Group-2 and Group-3: p = .000). However, no statistically significant differences were found between the groups reading printed texts and digital texts on a screen in any sub-dimension. Nevertheless, it can be stated that the group reading printed texts showed higher performance in reorganization, inferential comprehension, evaluation, and appreciation, whereas the group reading digital texts on a screen performed better in literal comprehension.

## 5. Discussion

This study investigated the effects of printed, digital, and augmented reality (AR)-supported texts on students’ reading comprehension performance. As reading materials, texts from students’ native language textbooks were chosen. Additionally, the digital versions of these texts, published by the Ministry of National Education, were used during the screen-reading process. Furthermore, AR-supported texts were prepared based on these printed textsThe main finding of the study is that AR-supported texts significantly improved students’ reading comprehension performance. Therefore, it can be concluded that AR books improve the reading comprehension performance of children aged 11–12. This result indicates that AR applications can make a meaningful difference in learning environments [134,135]. However, there was no significant difference in reading comprehension performance between children engaged in traditional and screen-based reading.

When students’ reading comprehension scores were evaluated overall, no difference was observed in performance between the two groups participating in traditional and screen-based reading. Indeed, the results of some studies in the literature are similar to the findings of the present study [94,95,136–140]. Printed and digital texts are structurally the same, consisting of text and visuals. However, the only difference between these reading materials is that printed texts were read from a book, whereas digital texts were read on a computer. Hermena et al. [94] suggested that when conditions such as brightness and contrast are controlled during screen reading, comprehension levels can be similar to those of traditional reading. Within this framework, as observed in similar studies in the literature, the current study also found no significant difference in students’ reading comprehension performance between traditional and screen-based reading. However, when meta-analyses examining the effects of traditional and screen-based reading on comprehension are reviewed, printed texts generally emerge as more advantageous compared to digital texts [102,103,141,142]. Similarly, the study conducted by Salmerón et al. [143] found that most digital-based activities may hinder students’ reading development. The study explained that children might perceive interaction with digital tools as a leisure activity characterized by low effort and distraction, and this perception could trigger superficial attention and distracting behaviors in learning environments. This finding may suggest that students are unable to sufficiently focus their attention and cognitive resources during the digital reading process. Thus, the findings in the literature are inconsistent. While some studies, including the present study, reported no difference in children’s reading comprehension performance between printed and digital texts, other studies have found traditional reading to be more effective than screen reading [25,92,98,100–104,144,145]. Conversely, some research has indicated that screen-based reading is more effective than traditional reading [12,90,91,146–148]. Additionally, the study conducted by Tseng and Yeh [149] found that screen reading—especially when supported with personalized digital texts in an online environment—can enhance comprehension among low-achieving students. This suggests that the flexible nature of screen reading may accommodate different learning styles. In conclusion, although varying findings exist in the literature, the present study indicates no significant difference in reading comprehension performance between students participating in traditional reading and screen-based reading activities.

The study found no significant differences between the traditional reading and screen-based reading groups in any of the sub-dimensions of reading comprehension: literal comprehension, reorganization, inferential comprehension, evaluation, and appreciation. When examining the relevant literature, these results align with studies reporting no differences in reading comprehension between traditional and screen-based reading [92,150–152]. This finding indicates the necessity of investigating the impact of variables such as student age group, text structure, and test type on reading comprehension [153]. On the other hand, a study conducted by Chen et al. [154] compared the effects of tablet and computer interventions on reading comprehension performance with traditional reading. The results showed that the group reading printed texts performed significantly better surface-level comprehension than computer-based reading. However, no differences were found between the two groups regarding deep comprehension. While this aspect of the results is consistent with the present study, the findings differ regarding surface-level comprehension. In a study by Singer and Alexander [92], students reading digital texts demonstrated higher prediction levels than those reading printed texts. Conversely, Duncan et al. [155] found that participants reading traditional texts outperformed those reading digital texts regarding inference-making and prediction. Therefore, inconsistencies exist in the results of studies in the literature when evaluating the sub-dimensions of reading comprehension, just as they do for overall comprehension scores. Additionally, the study conducted by Florit et al. [156] demonstrated that digital-based reading poses a disadvantage compared to paper-based reading. The study found that the navigational freedom offered by digital texts had a lower impact, particularly in solving inferential questions. This finding suggests that the disruption of structural coherence on the screen may hinder the process of comprehension. Additionally, while some studies have shown that readers of printed texts tend to spend more time and take more notes than readers of digital texts, they also reported that the differences in reading comprehension performance between the two groups were insignificant [134]. In conclusion, the present study revealed similar results across all sub-dimensions of reading comprehension.

The findings related to the first research question, “What is the difference in reading comprehension scores among the groups reading printed, digital, and augmented reality-supported texts?” revealed that students exposed to the AR intervention demonstrated higher reading comprehension performance compared to those who engaged with printed and digital texts. This supports studies suggesting that the interactive, multisensory, and engaging learning environments provided by AR technology can more effectively stimulate students’ cognitive processes [117,157,158]. This finding makes a significant contribution to the literature by indicating that AR can particularly support the cognitive development of children aged 11–12. Indeed, recent studies also show that AR-supported materials can enhance students’ abilities to access, comprehend, and retain information [50,117,159,160]. Examining the relevant literature reveals that studies have frequently focused on comparing AR storybooks with printed texts in the context of reading comprehension performance. This study is significant as it presents the results of a multi-dimensional comparison that includes screen reading. The findings indicate that, among the three groups, the AR intervention was the most effective in enhancing reading comprehension performance. When examining related studies in the literature, the results of the study conducted by Çetinkaya Özdemir and Akyol [75], which investigated AR-supported reading activities, align with the present study’s findings. Their results showed that the AR intervention provided more significant support for students’ reading comprehension performance than traditional reading activities. Similarly, the study by Xie, Gong, Qu & Bao [160] found that students participating in reading activities using AR materials outperformed those using traditional reading methods in terms of comprehension. Although a small number of studies have reported less positive results in this area [61,120], the majority of studies in the literature suggest that AR applications can improve students’ reading comprehension outcomes [4,35,62,63,66,161,162]. In this context, the ability of AR books to provide students with interactive experiences and enrich the learning process [69] may have contributed to the improvement in reading comprehension performance. Additionally, AR’s advantages in enhancing motivation, engagement, creativity, imagination, and collaboration [120,163–165] could also have played a role in this improvement. Moreover, the dual advantages of AR storybooks—providing the tactile experience of a physical book while incorporating the multisensory features of digital books [117,166–168] —may have further supported users’ reading comprehension performance.

One notable aspect of the present study is that reading comprehension was not evaluated as a single dimension. Many augmented reality(AR) studies have considered reading comprehension a general concept [75,158,169]. Barrett’s Taxonomy was utilized in this study, and reading comprehension was examined across five sub-dimensions. This Barrett Taxonomy-based approach is one of the few studies in the literature that examines in detail the different cognitive effects of AR-supported reading [62,170]. In this respect, the study makes a methodological contribution to the field. Regarding the second research question—“What are the differences in literal comprehension, reorganization, inferential comprehension, evaluation, and reaction scores among the groups reading printed, digital, and augmented reality-supported texts?”—the findings revealed that children who read the AR book performed better than those in other groups across all dimensions: literal comprehension, reorganization, inferential comprehension, evaluation, and reaction.

These results indicate that analyses based on Barrett’s Taxonomy are important for evaluating reading comprehension performance in a multidimensional way and that AR-supported materials can be an effective tool in developing higher-order cognitive skills. In this regard, the present study distinguishes itself from many others in the literature by examining not only overall achievement scores but also the specific components of reading comprehension individually. A literature review indicates that similar results have been found in other studies. In line with the results of the current study, the research conducted by Bursalı and Yılmaz [171] found that AR books support higher-order comprehension skills and enable students to engage with the text for a longer period. For example, Liu et al. [63] examined the effects of AR picture books on the reading comprehension of elementary school students and found that AR picture books were more effective in enhancing participants’ performance on implicit questions during comprehension tests. In a similar vein, the study conducted by Şimşek and Direkci [74] demonstrated that, compared to printed texts, AR-supported reading practices enhanced students’ performance on questions requiring the inference of implicit meanings based on prior knowledge and contextual cues. Similarly, Danaei et al. [65] reported that children participating in AR-supported reading activities were more successful in answering implicit questions requiring prior knowledge and inference than children engaged in traditional reading activities. In line with the findings of the present study, Ebadi and Ashrafabadi [67] revealed that AR content not only enhances students’ comprehension but also supports their reading skills by providing rich background knowledge, an interactive environment, and reducing cognitive load. Accordingly, it was concluded that AR-supported content offers multifaceted contributions to the learning process and serves as an effective tool, particularly in the development of reading comprehension skills. These results indicate that the pedagogically grounded integration of AR storybooks—one of today’s prominent educational technologies—can significantly enhance meaningful learning, especially within interactive learning environments [162,166]. However, there are a few studies whose findings are inconsistent with the results of the present study. For example, Tobar-Muñoz et al. [61] found no difference in the recall performance of factual and inferential questions between a group reading books using an ARGBL game and a group reading traditional books. This discrepancy may be attributed to using a more complex game in the study, which may have imposed a more significant cognitive load on the experimental group. Additionally, some studies have identified the negative effects of excessive digitalization on students [161]. Poor integration of AR content during learning can lead to biases against multimedia content and abandonment of digital materials by students [162]. Currently, there are no established design principles for preparing AR content. Therefore, different digital elements are integrated into texts during AR storybook development, which may account for the variability in results across studies. Moreover, studies emphasize that for AR applications to be effective, a balance between content and cognitive load must be carefully considered. Researchers note that rather than enhancing comprehension, AR materials may become distracting—especially in designs that are overly interactive [172,173]. For instance, the study by Şimşek and Direkci [74] found significant differences in favour of the AR group in the reorganization, inferential comprehension, evaluation, and appreciation sub-dimensions when comparing students reading AR storybooks to those reading traditional books. However, no difference was observed between the groups regarding literal comprehension. This result differs from the present study despite using the same texts. The primary reason for this discrepancy may be the modification of AR content in Şimşek et al.’s [131] study, where AR content was integrated into the texts following specific design principles. These principles produced results that support children’s comprehension [131]. Thus, paying attention to specific design principles when preparing AR content can enhance reading comprehension performance.

In conclusion, this study demonstrates that augmented reality-supported texts can enhance students’ reading comprehension performance and provide meaningful contributions, particularly in the sub-dimensions of reading comprehension. In this regard, augmented reality applications can be considered an effective tool in educational settings aimed at developing reading skills.

## 6. Conclusion

This study examined the effects of printed, digital, and augmented reality (AR)-supported texts on students’ reading comprehension performance. Within this scope, the texts were first identified. These texts’ printed and digital versions were obtained, and an AR storybook was developed. A significant portion of previous studies in the literature has compared the effects of AR storybooks only with printed texts. In this regard, including digital texts in this study provides the literature with updated data. During the research process, after preparing the texts, students’ reading comprehension levels were assessed using a pre-test and post-test administered through an achievement test. The comprehension performance of the groups was evaluated based on the five levels of comprehension outlined in Barrett’s Taxonomy. Most studies on reading comprehension in the literature evaluate participants’ comprehension scores on a single dimension. Therefore, this study provides an opportunity to assess the effects of the interventions on participants’ reading comprehension performance in a more detailed and nuanced manner.

The study’s results indicated no significant difference in reading comprehension performance between students who read printed and digital texts. This was also true for the sub-dimensions of literal comprehension, reorganization, inferential comprehension, evaluation, and appreciation. However, students reading the AR storybook demonstrated higher reading comprehension performance than the other groups. Similarly, the same result was observed across all sub-dimensions of reading comprehension. Thus, it can be concluded that AR storybooks are a supportive tool for enhancing children’s reading comprehension performance. In conclusion, the study’s findings suggest that AR storybooks can be considered materials suitable for use in school settings.

## 7. Limitations and recommendations

The present study has several limitations. First, this study was conducted with participants aged 11–12 years. Therefore, the findings are limited to this age group. Future studies could include participants from different age groups to contribute to the generalizability of the findings. The study used three texts from students’ textbooks, and potential differences arising from text genres were not addressed. Future research could focus on how these interventions affect comprehension across different text genres. The Ministry of National Education published the printed and digital texts used in the present study. Therefore, no modifications were made to the texts. However, digital texts could be designed with additional content, which might influence the results. Similarly, the AR storybook could be enriched with interactive options and 3D visuals, potentially altering the outcomes. Future studies could examine the effects of such enriched content on reading comprehension. This study evaluated children’s reading comprehension performance across five dimensions based on Barrett’s Taxonomy, providing a more detailed perspective. It is recommended that similar studies in the literature also assess participants’ reading comprehension performance not only through total scores but also in terms of sub-dimensions such as “literal comprehension,” “inference-making,” and “evaluation.” This approach could enable more detailed comparisons across studies and yield more nuanced findings.
